# The impact of type 2 diabetes on aging: multidimensional approaches to preserve cognitive health

**DOI:** 10.1007/s00592-025-02583-3

**Published:** 2025-09-02

**Authors:** Stefania Lucia, Silvia Fornaro, Massimo Federici, Raffaella Ida Rumiati

**Affiliations:** 1 Neuroscience Area, Scuola Internazionale Superiore di Studi Avanzati (SISSA), Trieste, Italy; 2 Department of Systems Medicine, University of Roma Tor Vergata, 00133 Rome, Italy

**Keywords:** Type 2 diabetes, Aging, Cognition, Semaglutide, Physical activity

## Abstract

The growing prevalence of type 2 diabetes (T2D) among older adults represents a major public health concern, given its association with accelerated cognitive decline and increased risk of neurodegenerative diseases. Several diabetes-related mechanisms, including chronic hyperglycaemia, oxidative stress, vascular dysfunction, and insulin resistance in the brain, negatively impact key cognitive domains, including memory and executive functions. These neuropathophysiological alterations are also linked to structural brain changes, contributing to vulnerability to dementia. This narrative review examines both established and emerging strategies aimed at counteracting the cognitive impact of T2D in aging populations. Traditional interventions, especially structured physical activity programs, have consistently demonstrated benefits for global cognitive functioning. In parallel, new pharmacological treatments, such as GLP-1 receptor agonists (e.g., semaglutide), not only improve glycemic control but may also exert neuroprotective effects. Multidomain approaches integrating metabolic management, nutritional optimization, physical exercise, and social engagement, such as those tested in the J-MIND-Diabetes study, have yielded promising outcomes in preserving cognitive functions. We argue that combining pharmacological and behavioral strategies holds significant potential for supporting cognitive health in elderly individuals with T2D. Such multimodal interventions may enhance resilience to cognitive decline, improve quality of life, and promote healthy brain aging in this at-risk population.

## Introduction

The global aging population, projected to exceed 2.1 billion individuals over the age of 65 by 2050, presents a major challenge for healthcare systems worldwide. In Italy, the demographic shift toward an older population poses one of the greatest pressures on the National Health Service, both in terms of care provision and economic sustainability. Among the chronic conditions affecting older adults, type 2 diabetes (T2D) stands out due to its increasing prevalence and the substantial burden it places on healthcare systems, individual autonomy, and quality of life.

T2D is a chronic metabolic disorder caused by a combination of insulin resistance and impaired insulin secretion. According to the diagnostic criteria established by the American Diabetes Association [1] and the World Health Organization [2], T2D is defined by fasting plasma glucose ≥ 126 mg/dL, HbA1c ≥ 6.5%, or a 2-hour post-load glucose ≥ 200 mg/dL during an oral glucose tolerance test. It is strongly associated with modifiable risk factors, such as obesity, sedentary lifestyle, and unhealthy diet, as well as non-modifiable ones like aging and genetic predisposition. Recent estimates indicate that approximately 2.8% of national health expenditure in Italy is attributable to T2D, with an economic impact of nearly $58 billion annually [3, 4].

While both type 1 and type 2 diabetes are characterised by hyperglycaemia, their pathophysiological mechanisms differ markedly. This review specifically focuses on T2D, the most common form among older adults, and increasingly associated with accelerated cognitive impairment and a higher risk of neurodegenerative diseases.

Given this dual burden, metabolic and cognitive, understanding and managing T2D in elderly individuals is critical not only for achieving glycaemic control but also for preserving cognitive health and promoting active and independent aging. This review explores the neurobiological mechanisms linking T2D and cognition and critically evaluates emerging multidomain strategies including lifestyle interventions, pharmacological treatments, and their integration, for supporting brain health in this vulnerable population. Notably, although the present review is narrative in nature, we adopted a specific search strategy using Scopus to identify relevant studies. The search string included the following terms: ( (“type 2” OR “type-2“ OR “type two”) AND ( diabet*) AND ( age* OR elder* OR old*) AND (treat* OR medication*) AND ( brain* OR cognit*). We considered only studies published in the last 20 years (2005–2025) to ensure an updated overview of the topic. While we did not apply strict inclusion and exclusion criteria, the authors carefully screened the literature, to select original studies meeting high standards in original studies either in theoretical and methodological rigor.

## The metabolic characteristics of T2D

T2D is characterised by a complex metabolic dysfunction, primarily involving insulin resistance and a relative deficit in insulin secretion. Insulin resistance manifests itself as a reduced ability of peripheral tissues (skeletal muscle, liver and adipose tissue) to respond to insulin. In muscles, this results in reduced glucose uptake, while in the liver it leads to excessive glucose production (gluconeogenesis) despite high insulin levels. In adipose tissue, insulin resistance promotes increased lipolysis, resulting in the release of free fatty acids into the bloodstream, which further contributes to dyslipidaemia and ectopic lipid accumulation in organs such as the liver and muscles, exacerbating insulin resistance [5, 6].

In parallel, pancreatic beta cells show a functional deficit, with a reduced ability to secrete insulin in response to hyperglycaemia. This deficit is progressive, often worsening over time, leading to relative insulin deficiency. Furthermore, T2D isassociated with impaired secretion of incretins, GLP-1 (Glucagon-like peptide-1) that normally enhance insulin secretion in response to meals. The attenuation of incretins contributes to further beta-cell dysfunction [7].

T2D is also characterised by a chronic low-grade inflammatory state and increased oxidative stress, both of which play a central role in perpetuating insulin resistance and beta-cell failure, while contributing to systemic tissue damage [8].

Together, these pathophysiological mechanisms not only sustain chronic hyperglycaemia, but also account for the frequent association of T2D with obesity, arterial hypertension, and atherogenic dyslipidaemia, which significantly increase the risk of cardiovascular and neurocognitive complications.

## Neural and structural brain changes

Beyond systemic metabolic dysfunction, T2D exerts profound effects on the central nervous system, leading to alterations in both the structure and function of the brain. These changes, occurring at both the molecular and macroscopic levels, are increasingly recognised as key contributors to the cognitive deficits observed in individuals with T2D. At the microscopic level, synaptic integrity is particularly compromised. Insulin resistance in the brain impairs synaptic plasticity, the capacity of neurons to form and modify synaptic connections, an essential process for learning and memory [9, 10]. Chronic hyperglycaemia and oxidative stress exacerbate this disruption by damaging synaptic proteins and lipids, resulting in reduced synaptic density, especially in the hippocampus, a region crucial for episodic memory and spatial navigation [11]. T2D is also associated with imbalances in key neurotransmitters, such as glutamate and GABA, which impair excitatory/inhibitory signalling and compromise memory-related circuits [12].

At the macroscopic level, neuroimaging studies have revealed volume reductions in regions including the hippocampus, prefrontal cortex, anterior cingulate cortex, and temporal lobes—areas known to support memory, executive function, attention, and language [13, 14]. Additionally, T2D is associated with white matter lesions, particularly in periventricular areas, which reflect chronic vascular damage. These lesions have been linked to impaired processing speed, attentional control, and executive dysfunction, likely due to the disruption of long-range white matter tracts such as the fronto-striatal circuits, inferior fronto-occipital fasciculus, and corpus callosum [15].

Collectively, these neurobiological alterations, ranging from synaptic dysfunction to regional atrophy and disconnection, establish a pathological substrate that increases the brain’s vulnerability to cognitive decline and neurodegeneration. They also contribute to reduced neurogenesis and the accumulation of amyloid-β, a hallmark of Alzheimer’s disease [9, 11]. For a summary see Table 1.

**Table 1:** Pathophysiological mechanisms of T2D and brain impact

Mechanism	Brain/Cognitive Impact	References
Insulin resistance	Impaired synaptic plasticity, reduced neuronal insulin signaling	DeFronzo, 2009; De Felice & Ferreira, 2014
Chronic hyperglycaemia	Cerebral microangiopathy, oxidative stress	Arnold et al., 2018
Inflammation	Neuronal damage, cognitive decline	Hotamisligil, 2006
Reduced incretin secretion (GLP-1)	Worsened insulin secretion, potential neurodegeneration	Drucker, 2018
Hypoglycaemic episodes	Hippocampal damage, memory deficits	Whitmer et al., 2009

## Cognitive impairments

Consistent with the neural alterations described above, individuals with T2D frequently exhibit cognitive impairments across several domains, including episodic memory, working memory, attention, processing speed, and executive function. Deficits in episodic and working memory have been consistently reported and are particularly relevant given their overlap with age-related cognitive decline and early stages of dementia [16].

T2D has also been associated with impairments in attentional control and information processing speed. Palta and colleagues [17] found that diabetic patients demonstrated reduced ability to maintain focus and process information efficiently, resulting in difficulties with multitasking and rapid decision-making. Likewise, executive functions, including planning, cognitive flexibility, and inhibitory control, are often compromised, affecting complex goal-directed behaviour and adaptive responses [18].

Importantly, several studies have shown that individuals with T2D have a significantly higher risk of developing mild cognitive impairment (MCI) and dementia, including Alzheimer’s disease, compared to age-matched controls [12]. The underlying mechanisms are multifactorial: chronic hyperglycaemia contributes to micro- and macrovascular damage in the brain, promoting ischemia and disrupting perfusion; oxidative stress and systemic inflammation accelerate synaptic degradation and neuronal loss [9].

Moreover, insulin resistance interferes with brain insulin signaling, impairing memory consolidation and favouring the aggregation of neurotoxic proteins such as beta-amyloid [11]. Finally, recurrent hypoglycaemic episodes, particularly in patients on insulin therapy, may damage memory-related structures, such as the hippocampus, further contributing to neurocognitive dysfunction [19]. To a recapitulation see Table 2.

**Table 2:** Cognitive domains impaired in T2D

Cognitive Domain	Description of Deficit	Cognitive Tests Used	Sample Size	References
Episodic Memory	Difficulty recalling specific events	Word List Recall, Story Recall	*n* = 13,351 review of multiple studies	Rawlings et al., 2019 Biessels et al., 2020
Working Memory	Reduced capacity to retain/manipulate information	Digit Span, n-back tasks	*n* = 13,351	Rawlings et al., 2019
Attention	Difficulties in focus and sustained attention	Stroop Test, Continuous Performance Task	*n* = 2,347	Palta et al., 2014
Processing Speed	Slower cognitive processing and reaction time	Digit Symbol Substitution, Trail Making Test A	*n* = 2,347	Palta et al., 2014
Executive Function	Reduced planning, flexibility, inhibitory control	Wisconsin Card Sorting Test, Trail Making Test B	~ 16 studies review of multiple studies	Cukierman-Yaffe et al., 2009 Biessels et al., 2020

## Standard treatments for T2D

The clinical management of T2D requires a comprehensive approach that goes beyond glycaemic control, aiming to prevent or mitigate long-term complications, including cognitive decline. In this section, we review the main categories of treatment behavioural, pharmacological, surgical, and emerging therapies with particular attention to their potential impact on brain health and ageing trajectories.

## Behavioural interventions

The clinical management of T2D is based on a multidimensional approach targeting not only glycaemic control, but also the prevention of systemic and neurocognitive comorbidities. Lifestyle modifications remain the first-line intervention, with consistent evidence supporting the role of healthy diet and regular physical activity in improving glucose and lipid metabolism, as well as psychological and cognitive well-being [20, 21].

Structured behavioural interventions have been shown to reduce the risk of cognitive decline and dementia, particularly in older adults, by modulating oxidative stress and systemic inflammation [22]. Among the most effective protocols are 12- to 24-week training programmes that combine aerobic (e.g., brisk walking, cycling) and resistance exercise (e.g., weight training, bodyweight exercises), with a frequency of 3–4 sessions per week and a minimum weekly volume of 180 min. These interventions significantly improve insulin sensitivity, lower HbA1c levels, and alleviate depressive symptoms in individuals with metabolic dysfunction [23].

In parallel, nutritional interventions based on high-quality dietary patterns, such as the Mediterranean diet, have shown beneficial effects in reducing the incidence of T2D and hypertension, particularly in vulnerable populations [24].

## Physical activity as a core behavioural and neuroprotective strategy

Clinical and Metabolic Benefits of Physical Activity. Regular physical activity represents one of the most effective and accessible interventions to improve metabolic health in patients with T2D. Numerous clinical studies show that exercise enhances insulin sensitivity, reduces plasma glucose levels and improves the lipid profile, with positive effects on HbA1c and weight control [25]. Beyond the metabolic domain, physical activity influences body composition, blood pressure, mood, and cardiovascular function [26].

Obesity is a major risk factor for T2D onset and progression. Visceral adipose tissue (VAT) is associated with insulin resistance, chronic low-grade inflammation, and metabolic dysfunction [27]. Hence, interventions should aim to reduce body weight while preserving lean muscle mass through sustainable exercise programs. Moreover, exercise is known for its neuroprotective effects: it stimulates hippocampal neurogenesis, enhances synaptic plasticity, and increases levels of brain-derived neurotrophic factor (BDNF), supporting cognitive function and memory [28]. These effects are especially beneficial for patients with T2D who are at heightened risk of cognitive decline.

Structured programs combining aerobic and resistance training have been linked to reduced risk of dementia, improved attention and executive function, and enhanced psychological well-being [29]. This is particularly relevant for elderly patients with multimorbidity, frailty, or preexisting cognitive impairment. Integrating regular physical activity into daily routines can help maintain balance, strength, and autonomy, and ultimately support cognitive health and quality of life.

Types and Doses of Physical Activity: Evidence-Based Recommendations. Physical activity refers to planned, structured, and repetitive movements aimed at improving physical fitness. It is typically classified by intensity, type, and duration. Intensity is defined using the metabolic equivalent (MET), the amount of oxygen consumed at rest: light (< 3 METs), moderate (3–6 METs), and vigorous (> 6 METs) [30]. Aerobic exercises (e.g., brisk walking, running, cycling) improve cardiovascular and muscular metabolism. A study by Winnick et al. [31] showed that one week of aerobic training improved whole-body insulin sensitivity through increased muscle insulin action. Resistance training (e.g., weights, bands, bodyweight) targets lean mass increase and glucose uptake. Castaneda et al. [32] reported improved HbA1c and strength in older adults with T2D after 16 weeks of resistance training.

Combined aerobic and resistance training yields greater benefits than either alone. Schwingshackl et al. [33] found greater improvements in HbA1c, glucose, lipid profiles, and blood pressure in patients following combined protocols. The American College of Sports Medicine and ADA recommend ≥ 150 min/week of moderate-intensity or ≥ 75 min/week of vigorous-intensity physical activity. Młynarska et al. [34] suggest 150–300 min/week of moderate activity spread over ≥ 3 days. De Paola et al. [35] propose 3 sessions/week of moderate aerobic exercise plus 2 sessions of tailored resistance training. A meta-analysis by Gallardo-Gómez et al. [36] identified ~ 1,100 MET-minutes/week as the optimal threshold for glycemic control, equivalent to ~ 36 min/day of brisk walking. Greater benefits were seen in those with poorer baseline glycemia.

Personalised and Everyday Physical Activity. Di Murro et al. [37] emphasise the importance of personalised physical activity approaches tailored to individual metabolic and functional profiles, moving beyond standardised protocols. This can help improve glycemic control and preserve β-cell function sustainably. In patients with comorbidities or physical limitations, non-structured daily activities such as walking, gardening, housework, or errands also contribute meaningfully to energy expenditure and metabolic improvements [38].

Such activities contribute not only to physical health but also to the concept of cognitive reserve, the brain’s capacity to withstand age-related and pathological decline [39]. Cognitive reserve builds over time through stimulating education, social engagement, and daily activities. Regular leisure and home-based activities support neuroplasticity, reduce systemic inflammation, and counter oxidative stress, mechanisms vital for preserving brain function. Promoting these activities can thus serve as a practical and sustainable strategy for improving cognitive reserve in elderly patients with T2D, aiding in the prevention of cognitive decline and supporting healthy, active ageing.

In summary, while the literature provides extensive evidence on optimal types, intensities, and durations of physical activity in T2D, the need for personalised programs emerges strongly. Integrating structured and daily physical activity tailored to individual characteristics supports not only metabolic health but also cognitive function and quality of life in older adults.

## Surgical interventions

In cases of severe obesity, bariatric surgery remains a valuable therapeutic option. Excess adiposity is strongly associated with insulin resistance and glycaemic dysregulation, and surgical intervention can lead to partial or complete remission of T2D [40]. Beyond metabolic benefits, bariatric procedures have additionally been linked to reduced inflammatory markersand, indirectly, with cognitive improvements inked to better metabolic profiles [22].

Large-scale studies report additional benefits, including increased eligibility for organ transplant lists, improved 10-year survival, and better cardiovascular outcomes [41]. Moreover, five years after surgery, overall healthcare expenditures are reduced by approximately 30%, particularly due to decreased medication costs [42].

## Adjunctive and emerging therapies

There is growing interest in synergistic and complementary strategies to enhance standard treatments. Preclinical studies indicate that combining GLP-1 receptor agonists (GPL-1 RAs) with mitochondria-targeting agents may promote energy expenditure, improve insulin sensitivity, and reduce the accumulation of neurotoxic proteins in the brain [43]. In parallel, several natural compounds and nutraceuticals are under investigation for their potential to affect glucose metabolism. Although clinical evidence remains limited, some promising candidates include vitamin C and folic acid. Supplementation with vitamin C (500–1000 mg/day for > 30 days) has been associated with reductions in HbA1c; even modest improvements (e.g., − 1%) are clinically meaningful, as they are associated with decreased risk of microvascular and macrovascular complications. Folic acid (vitamin B9) may also enhance insulin sensitivity through anti-inflammatory and antioxidant mechanisms [44].

## Use of injectable medications as a modern Pharmacological approach

From a pharmacological perspective, glucagon-like peptide-1 receptor agonists (GLP-1 RAs) such as semaglutide and liraglutide, represent one of the most promising therapeutic classes. Beyond their well-established effects on glycaemic control and weight reduction, recent evidence indicates that GLP-1 RAs exert neuroprotective actions through mechanisms such as improved blood–brain barrier integrity, reduced neuroinflammation, and enhanced synaptic plasticity [45]. These properties are particularly relevant for older patients with T2D, who are at elevated risk of cognitive impairment.

GLP-1 RAs have additionally been linked to a potential protective effect against Alzheimer’s disease, especially in individuals with metabolic comorbidities [46]. In addition, long-term treatment (≥ 5 years) with sodium-glucose co-transporter 2 (SGLT2) inhibitors has been shown to reduce systemic inflammation and improve insulin sensitivity. These benefits are reflected in lower C-reactive protein (CRP) levels and improvements in the triglyceride-to-HDL (TG/HDL) ratio, a key marker of insulin resistance [47].

In recent years, the therapeutic landscape of T2D has been profoundly transformed by the introduction of glucagon-like receptor agonists (GLP-1 RA), indicated for the treatment of T2D mellitus. Semaglutide is a drug approved in 2017 by the Food and Drug Administration (FDA) and is administered once a week via subcutaneous injection with the aim of stimulating the production of insulin by the pancreas, in order to reduce blood glucose levels. Semaglutide is one of the most prescribed drugs in the United States with over 9 million prescriptions in the last quarter of 2022 [48], of which 70% concerned the injectable form at a concentration of 2 mg per 1.5 mL [49] for the treatment of T2D and obesity.

Increasingly, drugs such as semaglutide are also proposed for body weight management, with dosages and trade names different from those indicated for diabetes, with the aim of obtaining significant results in terms of weight loss, cardiovascular protection and potential neuroprotective effects. In fact, semaglutide initially received much attention for its effectiveness in reducing body weight, which gave it extraordinary visibility outside of the clinical context, thanks to its off-label use for weight loss.

Celebrities and influencers on social media have promoted semaglutide as a rapid weight-loss solution, contributing to the viral spread of the hashtag #semaglutide, which surpassed 930 million views in June 2023 [50]. This surge in popularity has significantly increased demand leading to global shortages and reduced access for patients with metabolic diseases [51]. On social media, semaglutide is frequently portrayed as a “breakthrough” treatment for obesity, with more than one-third of the content emphasising cosmetic rather than medical use. Media enthusiasm, often fueled by dramatic bodily transformation narratives [52, 53], is reshaping public discourse around health, weight and therapeutic access, frequently overshadowing the drug’s intended clinical application.

Regarding clinical purposes, according to the meta-analysis by Wang et al. [54], the optimal dosage of semaglutide for the treatment of T2D is 0.5 mg or 1 mg once weekly, a dosage correlated with significant reductions in HbA1c and body weight. The review confirms the efficacy of these standard dosages compared to other GLP-1 agonists. In addition, the weekly formulation also proved to be advantageous in terms of cardiovascular safety and cost-effectiveness. semaglutide, administered once weekly, is a prime example of a new generation of therapies that not only regulate glucose metabolism but also address the systemic and metabolic complications of T2D. This multifactorial efficacy makes GLP-1 agonists a central component in modern pharmacological management of diabetes, especially in patients with obesity and high cardiovascular risk.

Recent research suggests that GLP-1 receptor agonists (GLP-1 RAs), semaglutide, may exert neuroprotective effects by modulating inflammation, oxidative stress, and insulin signaling in the brain. Specifically, Dou et al. [55] showed that GLP-1 RAs improve cognitive functioning in diabetics by acting on multiple levels: they reduce inflammatory stress, support synaptic plasticity, and promote neuronal survival by modulating intracellular mechanisms. Similarly, Gunturu [56] explored the use of these drugs in psychiatry, reporting possible antidepressant effects due to their action on the central nervous system. In fact, the author proposes that GLP-1 RAs may open a new therapeutic paradigm in psychiatry, offering benefits in disorders such as depression, schizophrenia, and bipolar disorder through mechanisms which work independently from metabolic regulation. A systematic review by Tempia Valenta et al. [57] confirms improvements in depressive symptoms, cognitive function, and a reduction in compulsive behaviors such as binge eating and substance abuse. Additionally, Hlavinka [58] highlighted the growing interest in the use of semaglutide in the management of severe psychological conditions, such as major depressive episodes or mood dysregulation, while highlighting the need for randomized clinical trials to verify these preliminary observations. Overall, these studies indicate that semaglutide may offer significant cognitive and psychological benefits, warranting further investigation of its use in both metabolic and mental health settings.

However, the increased use of these agents has also brought attention to some dermatological side effects. According to Chamberlin and Dabbs [59], gastrointestinal disorders are the most common side effects: nausea, vomiting, and diarrhea affect up to 30% of patients, especially during the early stages of therapy or after increasing the dosage. Fortunately, these symptoms tend to be transient and manageable with gradual titration, with a discontinuation rate limited to 3–4%. In addition, the review by Vambe et al. [60], reports the need to be alert to rarer but clinically significant adverse events, such as pancreatitis, hypersensitivity reactions, and a possible, although unconfirmed, risk of medullary thyroid cancer. In addition, adverse events such as injection site skin reactions, urticaria, bullous pemphigoid, and the “semaglutide face” effect, a media term indicating the loss of facial fat associated with the use of the drug, have been described [61]. These observations reinforce the importance of careful clinical monitoring, especially in patients with predisposing risk factors. In summary, GLP-1 RAs remain powerful and multifunctional therapeutic tools. However, clinical use must be accompanied by careful monitoring, taking into account both benefits and potential adverse effects, particularly in the dermatological and neurological contexts. Personalized patient management remains central to optimizing therapeutic outcomes and long-term safety. For a summary see Table 3.

**Table 3:** Overview of Pharmacological treatments in T2D and cognitive implications

Drug/Class	Mechanism of Action	Effects on Cognition	Side Effects
GLP-1 RAs (e.g., Semaglutide, Liraglutide)	Stimulate insulin secretion, reduce appetite, improve glycaemic control	Neuroprotection, improved memory and executive function, reduced neuroinflammation	Nausea, vomiting, injection site reactions, potential risk of thyroid C-cell tumors
SGLT2 Inhibitors	Increase glucose excretion via kidneys, reduce blood glucose and body weight	Indirect cognitive benefits via metabolic improvements	Genital infections, dehydration, possible risk of ketoacidosis
Adjunctive Therapies	Antioxidant and anti-inflammatory effects, improve insulin sensitivity	Potential cognitive protection through systemic effects	Generally well tolerated; high-dose vitamins may cause GI upset

## Potential confounding effects in Non-Clinical populations

Although GLP-1 receptor agonists (GLP-1 RAs) have shown promising neuroprotective and cognitive effects in individuals with T2D and related metabolic dysfunctions, the growing off-label use of these drugs among healthy individuals, particularly for weight loss, raises important interpretative concerns. Reported cognitive or psychological benefits in these populations may not reflect a direct pharmacological effect, but rather a confounding interaction with pre-existing advantages such as higher cognitive reserve, healthier lifestyle, and better access to medical resources. These individuals often combine pharmacological treatment with enhanced self-care, social support, and psychological motivation, all of which may independently contribute to improved cognitive performance and well-being. Therefore, future studies should carefully account for these baseline characteristics to avoid overestimating the cognitive-enhancing potential of GLP-1 RAs in non-clinical populations. Disentangling pharmacological effects from contextual and psychosocial factors is essential to clarify the true scope of these agents and to prevent misleading generalizations about their broader cognitive efficacy.

## The interaction between physical activity and semaglutide

As aging is accompanied by numerous physiological changes that increase vulnerability to chronic diseases such as T2D, which significantly impacts not only on metabolism but also on cognitive function and brain health, in the elderly, this is associated with a significantly higher risk of developing cognitive decline, vascular dementia and Alzheimer’s disease, further complicating therapeutic management and compromising functional autonomy [62]. Recently, promising evidence has emerged supporting the effectiveness of integrated strategies for the treatment for T2D, combining innovative pharmacological approaches, such as GLP-1 receptor agonists like semaglutide, with non-pharmacological interventions, including regular physical activity.

GLP-1 RAs, already known for metabolic and cardiovascular benefits, have also been shown to be effective in promoting synaptic plasticity, neurogenesis and neuronal protection, contributing to the reduction of inflammation and oxidative stress at the cerebral level [55, 63]. Cheng et al. [64] highlight how these drugs may have a neuroprotective role in patients with diabetes-related neurodegenerative disorders, such as Alzheimer’s disease and Parkinson’s. In addition, Romano et al. [65] have shown that exendin-4, a GLP 1 agonist, significantly improves cognitive function in elderly diabetic patients, regardless of glycemic control, probably through modulation of receptors in the hippocampus. At the same time, physical activity represents an accessible and well-tolerated means that improves insulin sensitivity, stimulates cerebral perfusion and mitigates age-related cognitive decline [66].

The interaction between the two approaches physical activity and GLP-1 RA could enhance neurocognitive benefits through converging neurobiological mechanisms [57, 67]. In this context, the therapeutic focus is on a combined management of the ageing brain, where metabolic well-being and mental health are strongly interconnected. A particularly relevant example of the effectiveness of a combined approach in the treatment of T2D is provided by Mensberg et al. [68], which examined the synergistic effects of the combination of supervised physical exercise and pharmacological treatment with liraglutide, a GLP-1 receptor agonist. The results showed that the integration of these two interventions led to a near-normalization of HbA1c, accompanied by significant reductions in body weight and blood pressure, compared with exercise alone. This approach has shown that the combination of behavioural and pharmacological interventions can yield greater benefits than their isolated administration, especially in elderly patients, where the multifactoriality of the disease requires integrated therapeutic strategies. The study also highlights the importance of considering personalisation of treatment, with emphasis on complementarity between physical activity and incretinian drugs to achieve more effective and sustainable metabolic control over time.

Other studies have demonstrated the potential of combining semaglutide and non-pharmacological interventions in elderly patients with T2D to enhance the effectiveness of the integrated approach. In particular, Ingersen et al. [69] showed that the association between supervised aerobic training and semaglutide treatment leads to a significantly higher improvement in secretory function of pancreatic β-cells than each isolated intervention. This suggests a physiological synergy between metabolic adaptation induced by physical activity and stimulation of the GLP-1 receptor, with substantial benefits on glycemic control. In parallel, the clinical trial of Cortes et al. [70] analyzes the effect of semaglutide combined with lifestyle counseling on changes in body composition, physical performance and biomarkers of brain ageing in individuals over 65 with insulin resistance. In this case, the interaction between pharmacological strategy and behavioral rehabilitation appears to be fundamental to counteract geriatric fragility.

Finally, the retrospective study by Fiore et al. [71] offers a “real-world” validation of these results, demonstrating that oral semaglutide improves glycemic profile, weight and cardiovascular index even in people over 65, with high tolerability. This evidence confirms that a multidimensional and personalized therapeutic model, combining semaglutide and physical activity promotion, represents an effective and sustainable solution to optimize clinical outcomes in the elderly with T2D. Specifically, in studies evaluating the multidimensional approach between physical activity and semaglutide, the typical protocol involved weekly injections starting at 0.25 mg in the first 4 weeks, followed by 0.5 mg until week 9, when the maintenance dose of 0.5-1 mg/week is reached [69, 70]. Immediately after, a 12-week supervised aerobic training program is introduced, structured in 3 weekly sessions of 45 min at about 75% of the maximum heart rate reserve.

Ingersen et al. [69] used just this sequence, starting with 20 weeks of semaglutide, followed by 12 weeks of exercise, achieving a significant enhancement of the β cell function compared to individual interventions. Instead, Cortes et al. [70] prescribes 20 weeks of semaglutide up to 1 mg weekly, supplemented with behavioral counseling based on diet and physical activity programs, in order to assess not only glycaemic control but also body composition, physical performance and biomarkers of ageing. Overall, this evidence provides practical guidance for clinicians indicating an administration of 8–12 weeks of semaglutide (0.25; 0.5-1 mg/week), followed by 12 weeks of targeted aerobic training (3 × 45 min). The present therapeutic guidelines may represent a multidimensional and replicable model to optimize glycemic control and physical and cognitive resilience in elderly with T2D.

This multimodaltherapeutic strategy also shows a significant impact on the neurocognitive well-being of the elderly population diagnosed with T2D. The multimodal approach combining semaglutide (GLP-1 RA) and regular physical activity helps to improve key cognitive domains such as episodic memory, selective attention and executive functions by acting through synergistic neurobiological mechanisms. Supervised aerobic exercise stimulates cerebral blood flow and the production of brain-derived neurotrophic factor (BDNF), essential for neuroplasticity and gray matter preservation, thus supporting the maintenance of working memory [57].

In parallel, epidemiological data on more than 130,000 patients indicate that semaglutide administration is associated with a 28% reduction in the risk of cognitive impairment compared to traditional oral antidiabetic drugs such as sitagliptin. Clinical studies also indicate that 16 weeks of GLP-1 RA treatment led to improvements in deferred memory, sustained attention and cognitive flexibility, especially in individuals over 65 assessed through the Mini-Mental State Examination (MMSE) and the Montreal Cognitive Assessment (MoCA). These improvements do not seem to be a consequence of glycemic control, but more likely of neurobiological processes involving hippocampal maintenance and dorsolateral prefrontal cortex activity, as evidenced in functional neuroimaging studies [67]. These effects are amplified when pharmacological treatment is associated with structured exercise programs, as demonstrated in recent trials on elderly adults with insulin resistance [70]. Lastly, this synergistic therapeutic model not only optimizes glycemic control, but also supports cognitive resilience and counteracts age-related brain decline, positioning itself as an effective and sustainable paradigm in the management of geriatric T2D.

In summary, in T2D management for elderly people, integration between GLP 1 receptor agonists (semaglutide) and supervised physical activity emerges as an effective, sustainable and multidimensional therapeutic strategy. This integrated approach has proved to be useful not only in improving glycemic control, body composition and cardiovascular index, but also to promote brain health and cognitive functions in a population vulnerable to neurodegenerative disorders and age-related cognitive decline. Therefore, it represents a concrete and replicable solution to the need for integrated interventions to optimize metabolic, physical and cognitive resilience in elderly patients with T2D.

### Conclusions and future directions

T2D represents one of the main challenges of geriatric medicine, as a chronic condition that has a profound impact on the daily functioning, autonomy and quality of life of elderly people. The increased risk of cognitive decline, vascular dementia and Alzheimer’s disease in diabetic subjects makes the development and application of multidimensional therapeutic approaches and guidelines even more urgent. In this scenario, the combination of pharmacological treatment with GLP-1 receptor agonists and non-pharmacological interventions such as supervised physical activity is an integrated, effective and sustainable strategy. Alongside the metabolic and cardiovascular benefits, this therapeutic synergy has shown promising effects on brain health and cognitive function.

**Fig. 1:**
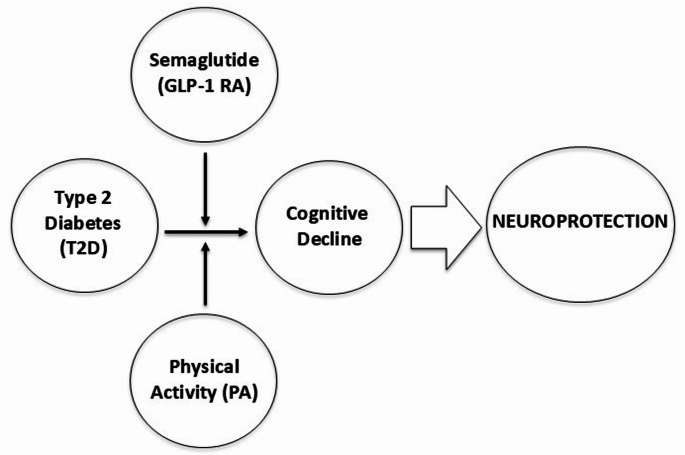
Integrated Neuroprotection Strategies in Type 2 Diabetes. PA and semaglutide may modulate the risk of cognitive impairment in subjects with T2D, contributing to an overall neuroprotective effect

Particularly relevant is the potential of such interventions to stimulate neuroplasticity, preserving the hippocampus and the prefrontal cortex from a structural and functional viewpoint, through the combined action of physical exercise and GLP-1 RAs. Specifically, this combined therapeutic approach could delay the onset of clinical symptoms related to cognitive decline. In turn, this could significantly prolong the functional autonomy of elderly people, by preventing the deterioration of key cognitive functions (e.g., memory, decision making), whose preservation is fundamental to perform everyday life tasks (see Fig. 1).

This approach thus helps to protect not only metabolic function but also the identity, independence and social participation of the elderly person. Notably, the latter are concepts closely linked to cognitive reserve, the ability of the brain to compensate for neuropathological damage through increased efficiency and flexibility of neural networks.

However, some important questions require further experimental investigation: the most effective protocols in terms of dosage, duration and timing of the therapeutic combination need to be defined more precisely, taking into account fragility, possible comorbidities and psychosocial barriers of the geriatric population. Conducting further randomized longitudinal studies is essential to assess the long-term effects on cognitive impairment risk, cognitive reserve evolution and prevention of geriatric syndromes.

At a time when the aging population is becoming increasingly important, promoting a multidimensional therapeutic paradigm means not only treating but also supporting the elderly person as a whole, enhancing their remaining resources and improving the trajectory of ageing.
